# An eHealth Project on Invasive Pneumococcal Disease: Comprehensive Evaluation of a Promotional Campaign

**DOI:** 10.2196/jmir.6205

**Published:** 2016-12-02

**Authors:** Donatella Panatto, Alexander Domnich, Roberto Gasparini, Paolo Bonanni, Giancarlo Icardi, Daniela Amicizia, Lucia Arata, Stefano Carozzo, Alessio Signori, Angela Bechini, Sara Boccalini

**Affiliations:** ^1^ Department of Health Sciences University of Genoa Genoa Italy; ^2^ Department of Health Sciences University of Florence Florence Italy

**Keywords:** invasive pneumococcal disease, pneumococcus, eHealth, mHealth, mobile app

## Abstract

**Background:**

The recently launched *Pneumo Rischio* eHealth project, which consists of an app, a website, and social networking activity, is aimed at increasing public awareness of invasive pneumococcal disease (IPD). The launch of this project was prompted by the inadequate awareness of IPD among both laypeople and health care workers, the heavy socioeconomic burden of IPD, and the far from optimal vaccination coverage in Italy, despite the availability of safe and effective vaccines.

**Objective:**

The objectives of our study were to analyze trends in *Pneumo Rischio* usage before and after a promotional campaign, to characterize its end users, and to assess its user-rated quality.

**Methods:**

At 7 months after launching *Pneumo Rischio*, we established a 4-month marketing campaign to promote the project. This intervention used various approaches and channels, including both traditional and digital marketing strategies. To highlight usage trends, we used different techniques of time series analysis and modeling, including a modified Mann-Kendall test, change-point detection, and segmented negative binomial regression of interrupted time series. Users were characterized in terms of demographics and IPD risk categories. Customer-rated quality was evaluated by means of a standardized tool in a sample of app users.

**Results:**

Over 1 year, the app was accessed by 9295 users and the website was accessed by 143,993 users, while the project’s Facebook page had 1216 fans. The promotional intervention was highly effective in increasing the daily number of users. In particular, the Mann-Kendall trend test revealed a significant (*P* ≤.01) increasing trend in both app and website users, while change-point detection analysis showed that the first significant change corresponded to the start of the promotional campaign. Regression analysis showed a significant immediate effect of the intervention, with a mean increase in daily numbers of users of 1562% (95% CI 456%-4870%) for the app and 620% (95% CI 176%-1777%) for the website. Similarly, the postintervention daily trend in the number of users was positive, with a relative increase of 0.9% (95% CI 0.0%-1.8%) for the app and 1.4% (95% CI 0.7%-2.1%) for the website. Demographics differed between app and website users and Facebook fans. A total of 69.15% (10,793/15,608) of users could be defined as being at risk of IPD, while 4729 users expressed intentions to ask their doctor for further information on IPD. The mean app quality score assigned by end users was approximately 79.5% (397/500).

**Conclusions:**

Despite its specific topic, *Pneumo Rischio* was accessed by a considerable number of users, who ranked it as a high-quality project. In order to reach their target populations, however, such projects should be promoted.

## Introduction

### Invasive Pneumococcal Disease: High Burden and Low Awareness

*Streptococcus pneumoniae*, also known as pneumococcus, is an important human pathogen. It can cause both noninvasive (eg, otitis media, sinusitis, pneumonia) and invasive pneumococcal disease (IPD), which is described as the presence of pneumococcus in normally sterile body fluids. IPD has a variety of clinical presentations, the most common being meningitis, bacteremia, and bacteremic pneumonia [1,2]. It has been estimated [3] that approximately 1.6 million people worldwide die of IPD each year. In Italy, IPD is the invasive bacterial infection that carries the highest burden in terms of morbidity. Indeed, in 2014, a total of 952 IPD cases were notified (accounting for 78% of all cases), while cases of invasive diseases caused by *Neisseria meningitidis* accounted for 163 (13%) and those caused by *Haemophilus influenzae* accounted for 105 (9%) [4]. The risk of developing IPD is unevenly distributed among different population groups, being significantly higher among young children, the elderly, and people with several underlying medical conditions and health-compromising behaviors [5,6].

Vaccination is the only public health measure able to drastically reduce the incidence of IPD [7,8] and is highly recommended [9] for the above-mentioned at-risk population groups. However, immunization rates remain relatively low in both Italy [9] and other developed countries, including the United States [10]. The reasons for this are very probably multiple and of different nature, although inadequate knowledge and awareness of IPD among both health care practitioners and patients seems to be a major factor. Indeed, Lode et al [11] found that the main obstacles to vaccination among laypeople were scant awareness of vaccine availability, insufficient IPD risk perception, and lack of recommendation by general practitioners (GPs). The same research group [11] reported scant awareness of terms for pneumococcal diseases, with only 50% of GPs knowing the term IPD. Nichol et al [12] found that recommendation by a health care provider enhanced pneumococcal vaccine adherence among adults at risk of IPD. A systematic review of the determinants of pneumococcal vaccination [13] confirmed this finding, revealing that strong recommendation by GPs is an effective strategy for increasing immunization rates.

### *Pneumo Rischio* Project

We have previously shown [14] that, despite the heavy health and socioeconomic burden of IPD in Italy [4], considerably less information is available on this disease than on other infectious pathologies with lower incidence rates. In order to fill this information gap—that is, the discrepancy between disease occurrence and available information [15]—we recently developed and launched an eHealth project called *Pneumo Rischio* [14]. Implementation of this project was also prompted by the above-described scant awareness of IPD among both laypeople and health care professionals [11-13] and the low vaccination coverage in Italy [9], despite the availability of safe and effective vaccines.

**Figure 1:**
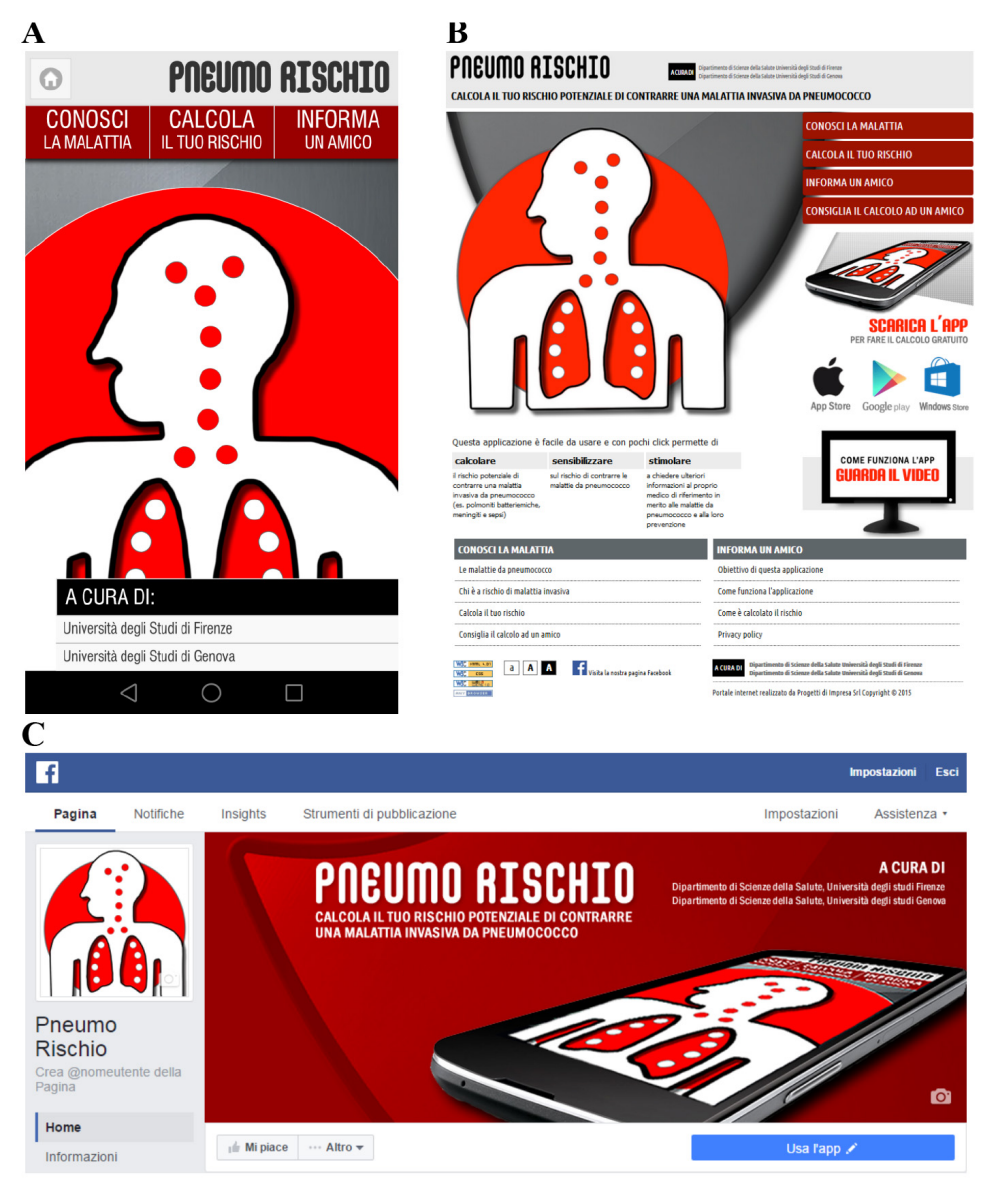
*Pneumo Rischio* app (A), website (B), and Facebook page (C).

*Pneumo Rischio* was launched on February 26, 2015 in the three main app stores available in Italy, namely Google Play (Google Inc, Mountain View, CA, USA) [16], iTunes (Apple Inc, Cupertino, CA, USA) [17], and Microsoft (Microsoft Corporation, Redmond, WA, USA) [18] stores. The development process and main features of *Pneumo Rischio* have been reported elsewhere [14]. Briefly, the app was conceived to be maximally functional and easy to use and navigate. The core component of the app is a checker, which is designed to estimate the personal risk of contracting IPD and inform its customers in a user-friendly manner of communication. Once users have completed the checker, they can send the complete output to their doctor by email. The ultimate goal of the app is to increase community awareness of IPD. We created a sister website [19] to ensure a higher population coverage [14]. Moreover, we have also set up a Facebook account (*Pneumo Rischio* product page, subcategory app page) [20] to share IPD-related information and resources. Figure 1 shows screenshots of the *Pneumo Rischio* app, website, and Facebook page.

### Rationale and Aims

To be effective, an app concerning health and health care should not only be technically efficient but also (1) be evidence based [21], (2) satisfy different aspects of objective and subjective quality [22], (3) be useful to and targeted at its end users [23], and (4) be downloadable and cover as much of the population as possible [24]. Undoubtedly, the first requirement should be satisfied in the initial stages of development; app developers, for example, could involve experts in a given health care field, employ methods of evidence-based medicine to build the product content, and test their app for quality and perceived usefulness in the phase of testing app prototypes. With regard to *Pneumo Rischio*, which proved to be a user-friendly product, we tried to make it evidence based by involving IPD experts in drafting content and features of the project and by carrying out a comprehensive literature review [14]. However, reaching this goal may not guarantee the achievement of the other three above-mentioned requirements. Thus, quality and usefulness evaluations made by real-world users after the app has been launched may differ from assessments made in the prelaunch phase, owing to poor representativeness of the target population or the fact that the judgments of real-world users may be scattered and highly subjective [22]. Continuous monitoring of users’ feedback and specific instruments may help assess user-perceived app quality and usefulness and may prompt modifications of the app content and functions in future updates. The fourth aspect (ie, the number of users) is of particular relevance to public health and preventive medicine.

In the light of the above considerations, we aimed to provide some potentially useful insights into assessing an app/eHealth project from different points of view. Specifically, with regard to the *Pneumo Rischio* project, our goals were (1) to describe and analyze the trend in usage and its determinants, (2) to characterize users and their representativeness, and (3) to evaluate users’ ratings of the quality of the app.

## Methods

### Marketing Campaign to Increase Population Exposure to *Pneumo Rischio*

In the first 7 months after the project was launched, we had no defined marketing strategy for its promotion; we only occasionally presented it at conferences, and we made some Web press releases. From the fourth week of September 2015, we established a more intense 4-month marketing campaign (henceforth referred to as the intervention). This used various approaches and channels, including both traditional and digital marketing strategies. The traditional approach consisted of presenting the app and the first results of its use at meetings, congresses [25,26], and postgraduate courses, participating in an eHealth competition, and advertising at physicians’ offices. The online component included online advertising (Google AdWords, Google AdSense, Google AdMob, Facebook Ads) and social media (primarily Facebook). While pay-per-click ads are a recognized and cost-effective means of advertising [27], social networking offers a unique opportunity for social promotion [28] and has proved to be a feasible recruitment option [29]. We used the *Pneumo Rischio* Facebook page to periodically post stylistically coherent messages concerning IPD risk factors. Most posts could be classified as designed questions [30] (eg, “Can my *Pneumo Rischio* increase if…?”). Moreover, we posted banners on some popular thematic and informational portals.

### Usage: Trends and Determinants

We used Google Analytics data, which are rigorously anonymous and presented in aggregated form, to record the number of users, number of sessions, users’ demographic characteristics, and the risk (related to IPD) profiles of *Pneumo Rischio* users. The main unit of analysis was the daily (n=365) number of app and website users. We considered app and website users separately, since they have different characteristics and usage patterns [14].

We exploited different techniques of time series analysis to highlight the usage trend. The modified Mann-Kendall test [31] for serially autocorrelated data was used to assess the statistical significance of the trend in the number of *Pneumo Rischio* users over time. We then performed change-point detection analysis to locate points at which the statistical properties (both mean and variance) of the time series changed [32]. For this purpose, we used the binary segmentation approach.

We subsequently carried out segmented negative binomial regression analysis (to account for overdispersion) of interrupted time series in order to quantify immediate and time-related changes in the counts of users after the launch of the intervention. Specifically, the equation was formulated as follows:

U_t_= *β*_0_+ *β*_1_×time_t_+ *β*_2_×intervention_t_+ *β*_3_×time postintervention_t_+ *ε*_t_,

where U_t_ is the mean number of app or website users on day *t*; *time* is the day from the *Pneumo Rischio* launch; *intervention* is a binary variable indicating time *t* before (0) or after (1) the start of the intervention; *postintervention* is the number of days after the start of the intervention on day *t*, expressed as 0 before the intervention and (time-detected change point) after the intervention; *β*_0_ is the baseline level of users at time 0; *β*_1_ is the day-by-day change in the mean number of users before the intervention; *β*_2_ is the level change in the mean number of users immediately after the intervention; *β*_3_ is the trend change in the day-by-day mean number of users after the intervention in comparison with the preintervention period; and *ε* is the error term [33]. Since residuals of both models were heteroskedastic and serially correlated, the inferential testing of model parameters used heteroskedasticity-autocorrelation consistent standard errors.

Subsequently, we described usage of the *Pneumo Rischio* Facebook product page; we collected these anonymous and aggregated data from Facebook Insights. We also investigated how different types of Facebook posts—ie, “Photos” (posts containing photos), “SharedVideo” (posts containing videos), “Notes” (posts with HTML capability and no word limit), and “Links” (posts with links to other sites)—can affect the engagement of visitors with the *Pneumo Rischio* Facebook page. Posts were also dichotomized by type into personalized (posts containing personal or possessive adjectives or pronouns; eg, “my,” “mine”) and neutral posts (without any personal determiners). Since the distribution of people’s engagement with Facebook posts was highly skewed, we applied the nonparametric Mann-Whitney *U* test and computed the effect size as Cohen *r*_c_= *z* /√ *n* [34].

### *Pneumo Rischio*: End Users’ Characteristics

Next, we characterized *Pneumo Rischio* users (app and website users and Facebook fans) in terms of sex, age, and IPD risk categories; that is, low (healthy adults), medium (healthy elderly and immunocompetent people of any age with chronic conditions), and high (immunocompromised people of any age) [35]. These data were collected from Google Analytics (app and website) and Facebook Insights (Facebook page). As a theoretical measure of the effectiveness of the project, *Pneumo Rischio* users were asked (once the final result had been visualized) whether they intended to ask their GP for further information on IPD and its prevention. The second proxy measure of project effectiveness was the number of emails with detailed IPD risk profiles sent. This proxy measure is based on the assumption that a user sends a personal health-related record to his or her GP.

### Objective and Subjective Quality as Defined by End Users

The quality of the app was assessed by means of the Mobile Application Rating Scale (MARS) [22], the Italian version of which has recently been validated [36]. The scale consists of 23 Likert-type items on a 5-point range (from 1, “poor,” to 5, “excellent”) and assesses app quality in 4 objective dimensions (engagement, functionality, aesthetics, and information) and 1 subjective quality dimension. A summary score for each dimension is obtained by averaging the corresponding scores of single items. The MARS total score is obtained by averaging the summary scores on the 4 objective quality dimensions. The Italian version of MARS has shown good psychometric properties; the MARS total score has been seen to have an intraclass correlation coefficient of .96, Cronbach alpha of .90, and acceptable levels of convergent, divergent, discriminative, known-groups validity, and scalability [36].

Since the MARS was originally intended to be used by trained professionals, a simplified training-free version of the scale was also created in order to obtain app-user quality ratings [37]. The two MARS versions are very similar; however, the user version uses simpler wording, contains fewer technical terms, and omits 3 items on the information subscale (accuracy of the app description in the app store, goals, and evidence base) [22,37]. Moreover, both MARS versions have an app-specific section that is adjustable to research aims. In this study, we evaluated the potential impact of the app on users’ knowledge, attitudes, awareness and behavior. Considering the above similarities, we supposed that most psychometric properties of the professional version would be transferable to the user version.

Subsequently, we assessed the customer-determined quality of the app. For this purpose, we enrolled participants during conferences and courses (health care professionals) and lectures (students in various graduate and postgraduate courses). Enrolled participants were instructed to navigate in all app components and functions for at least 10 minutes, to examine the description/definition of the app quality provided for each subscale, and then to fill in an anonymous paper-and-pencil survey form. Before evaluating the app, participants were not aware of the *Pneumo Rischio* project.

Participation in this nonbiomedical, noninterventional study was voluntary, and anonymity was guaranteed. Ethical approval for this study was not required, since it focused only on the quality evaluation of an existing service, which is freely available in the public domain, with no potential risks for participants. We collected no personal or sensitive data.

We express approximately normally distributed summary scores as means with standard deviations, and we summarized single Likert scale items as medians with interquartile ranges (IQRs). Average MARS summary scores exceeding 3.0 points (60%) were regarded as satisfactory [38]. We compared the MARS total scores between sexes and professions (health care vs non-health care) by means of *t* test, and calculated the Pearson *r* correlation coefficient between the score and the age of raters. The effect size for the *t* test was quantified by means of Cohen *d*. Finally, we constructed multivariable linear models (chosen by minimizing the corrected Akaike information criterion) with heteroskedasticity consistent standard errors to predict the MARS total and subscale scores.

All statistical analyses and modeling procedures were performed in R environment (R Foundation for Statistical Computing).

**Figure 2:**
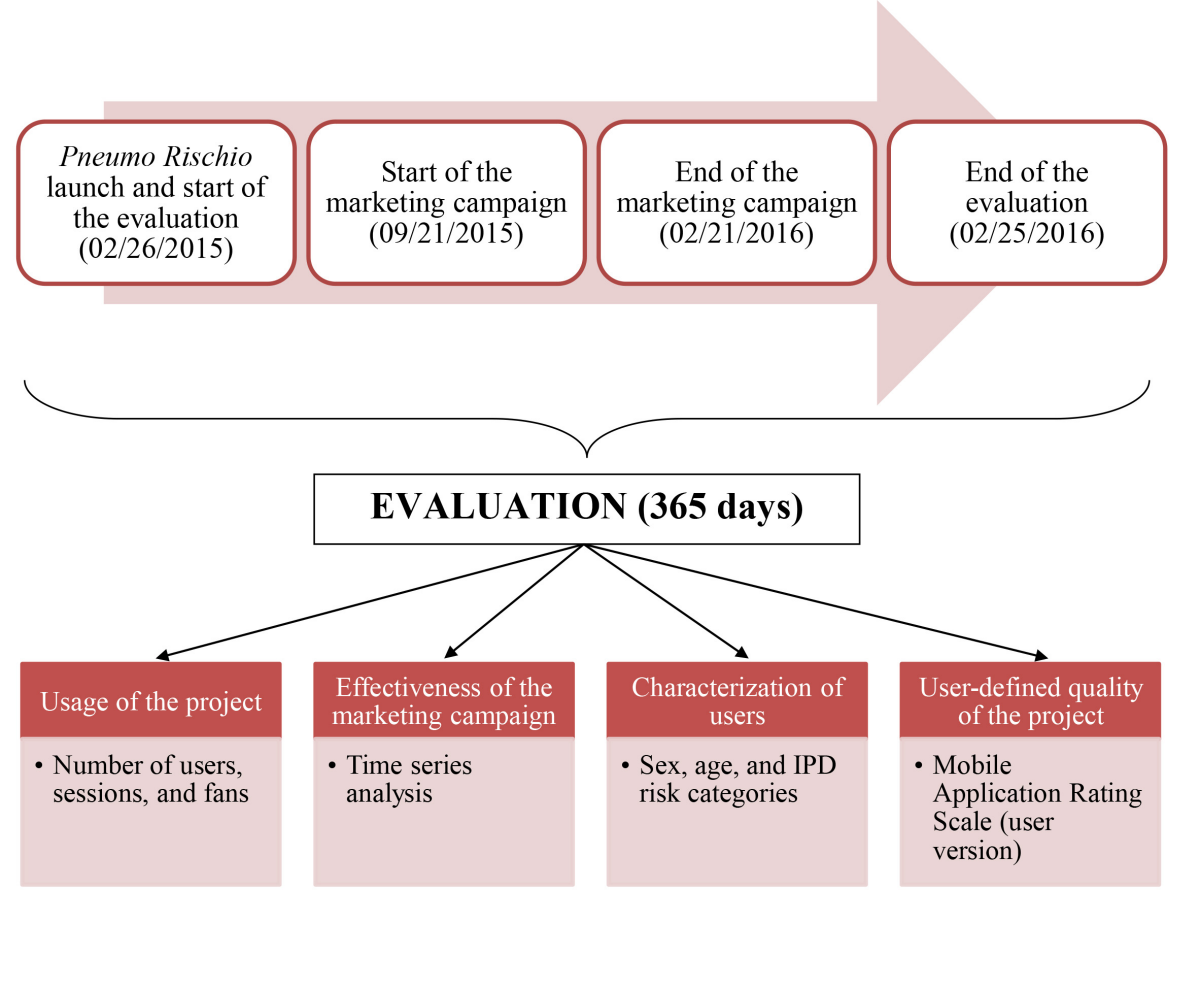
Chronology and assessment methods of the study of usage of the *Pneumo Rischio* project. IPD: invasive pneumococcal disease.

Figure 2 schematizes the chronology and assessment methods of the study.

## Results

### Usage: Trends and Determinants

On February 25, 2016, exactly 1 year after being launched, the app had been downloaded 9295 times and 10,090 sessions were run (1.09 sessions/user). On average, a session lasted 04:01 minutes. The website had been visited by 143,993 users, who ran 150,790 sessions (1.05 sessions/user), with a mean number of 2.13 pages per session. Website sessions lasted approximately four times less (01:02 minutes) than an average app session.

**Figure 3:**
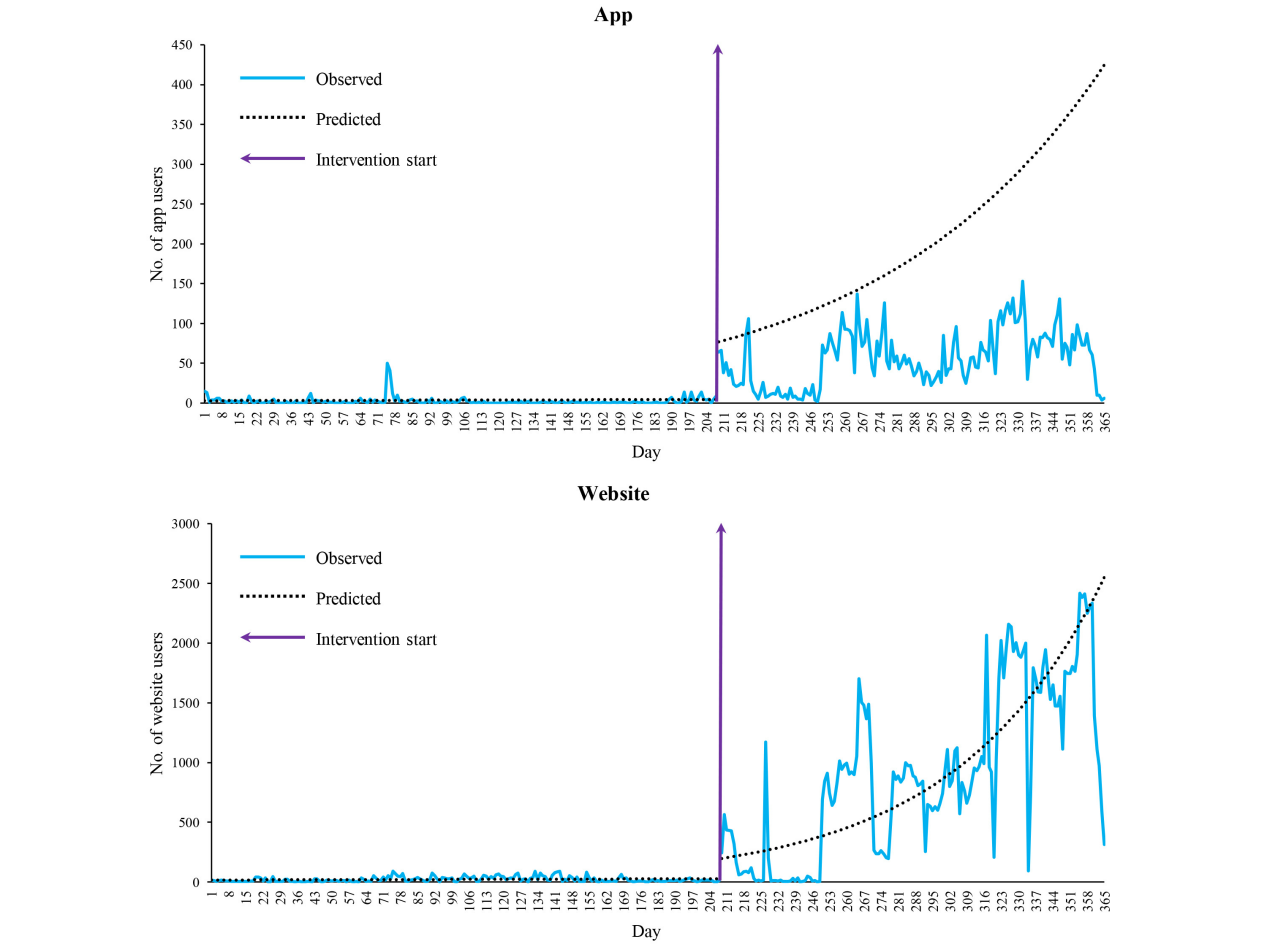
Daily observed and predicted numbers of *Pneumo Rischio* app and website users, February 26, 2015 to February 25, 2016.

The modified Mann-Kendall trend test showed a statistically significant increasing trend in both app (corrected *z*=3.33, *P*<.001) and website (corrected *z*=2.75, *P*=.01) users. Change-point detection analysis revealed 5 significant changes in the daily time series of both app and website use. The first point corresponded to the start of the intervention (fourth week of September 2015). Analogously, segmented regression analysis confirmed the effectiveness of the intervention (Figure 3). As Table 1 shows, the baseline level of app and website users was statistically different from zero. The preintervention trends displayed opposite patterns, being negative for the app and positive for the website; these parameter estimates did not, however, reach an alpha <.05. Estimated usage grew significantly (*P*<.001) immediately after the start of the intervention: we estimated an increase in daily numbers users of 1562% (95% CI 456%-4870%) for the app and 620% (95% CI 176%-1777%) for the website. Moreover, the postintervention day-by-day trend in the number of users was also positive and statistically significant for the website, with a 1.4% (95% CI 0.7%-2.1%) increase, but not for the app, with only a 0.9% (95% CI 0.0%-1.8%) increase.

On February 25, 2016, the *Pneumo Rischio* Facebook page had 1216 likes registered. The daily number of new likes correlated highly with both app (*r*=.60, 95% CI .53-.66) and website users (*r*=.59, 95% CI .52-.65). Since the start of the intervention, 30 posts had been published; most of these were of the Notes (n=16) and Photos (n=12) types, while there was only 1 SharedVideo and 1 Link. We excluded the SharedVideo and Link types of posts from the analysis, owing to their singularity. We categorized 9 posts as personalized, and the remaining 19 as neutral. Photo posts engaged a significantly (*P*<.001) higher median number of users than Notes (1075 vs 376), and the effect size was large (*r*_c_=0.74). By contrast, the higher number of users with personalized (median 631) rather than neutral (median 540) posts was not statistically significant (*P*=.76).

**Table 1:** Segmented regression analysis to predict the number of app and website users.

Parameter	App		Website	
	*β* (SE)	*z* (*P* value)	*β* (SE)	*z* (*P* value)
Baseline level	1.091 (0.449)	2.43 (.02)	2.804 (0.256)	10.95 (<.001)
Baseline trend	–0.002 (0.004)	–0.54 (.59)	0.002 (0.002)	1.10 (.27)
Postintervention level change	2.811 (0.559)	5.03 (<.001)	1.973 (0.489)	4.03 (<.001)
Postintervention trend change	0.009 (0.005)	1.86 (.06)	0.014 (0.004)	3.91 (<.001)

### *Pneumo Rischio*: End Users’ Characteristics

Demographic profiles differed between app users, website visitors, and Facebook fans (Table 2). In terms of the sex of users, women downloaded the app 1.5 times more frequently than men, and three-quarters of Facebook fans were female.

Conversely, a higher number of men visited the website. The most numerous age class of both app and website users was that of adults aged 25-34 years. *Pneumo Rischio* Facebook fans were older than *Pneumo Rischio* users, the most representative age class being 45-54 years, followed by the 55-64 and 35-44 age classes. It is encouraging that approximately 12% (145/1216) of the Facebook fans were aged ≥65 years.

**Figure 4:**
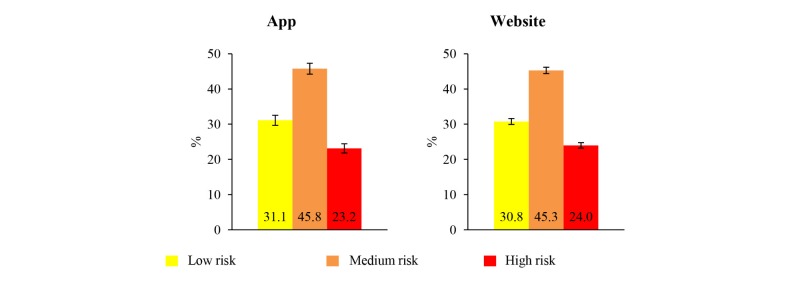
Distribution of risk categories for invasive pneumococcal disease among *Pneumo Rischio* app and website users (95% CI).

Approximately 70% (app: 2732/3965; website: 8061/11,643) of users may be defined as being at risk of IPD. The distribution of risk categories was similar between app and website users (Figure 4). A total of 2617 app users (25.9%, 95% CI 25.1%-26.8% of all sessions) and 2112 website users (1.4%, 95% CI 1.3%-1.5% of all sessions) stated that they would ask their GP for further information on IPD and its prevention. A total of 2142 (1700 for the app and 442 for the website) emails with detailed IPD profiles were sent.

**Table 2:** Age and sex distribution of *Pneumo Rischio* users.

Parameter		App users		Website users	Facebook fans	
		%	n/total	%^a^	%	n/total
**Sex**						
	Male	39.2	166/423	54.1	24.84	302/1216
	Female	60.8	257/423	45.9	74.92	911/1216
	Unknown	–		–	0.25	3/1216
**Age class, years**						
	18-24	7.0	29/412	27.5	3.13	38/1216
	25-34	29.9	123/412	33.5	8.63	105/1216
	35-44	23.8	98/412	15.5	23.11	281/1216
	45-54	18.2	75/412	12.5	29.20	355/1216
	55-64	13.6	56/412	5.5	24.01	292/1216
	≥65	7.5	31/412	5.5	11.92	145/1216

^a^Only relative data were available.

### Objective and Subjective Quality of the App as Defined by End Users

A total of 171 participants filled in the MARS user version. The mean age of participants was 34.3 (SD 12.7) years; females were more numerous (107/171, 64.1% women vs 60/171, 35.9% men). A total of 117 (68.4%) respondents were health care professionals. The mean MARS total score was 3.97 (SD 0.45), corresponding to 79.5%.

The mean scores on all MARS subscales exceeded the prespecified threshold of 60%, ranging from 62.8% for the subjective quality subscale to 84.4% for the functionality subscale. With regard to single items, most (15/20) had median scores of 4.0 points, while the median scores were 3.0 points for 4 items and 5.0 points for 1 item (Table 3).

**Table 3:** Mobile Application Rating Scale mean subscale and median item scores.

Subscale	Subscale scores			Item	Item scores	
	Mean	%	SD		Median	IQR^a^
Engagement	3.75	75.0	0.46	Entertainment	4	3-4
				Interest	4	3-4
				Customization	3	3-3
				Interactivity	3	3-4
				Target group	4	4-5
Functionality	4.22	84.4	0.55	Performance	4	4-5
				Ease of use	5	4-5
				Navigation	4	4-5
				Gestural design	4	4-5
Aesthetics	3.79	75.9	0.55	Layout	4	4-5
				Graphics	4	3-4
				Visual appeal	4	3-4
Information	4.12	82.3	0.51	Quality of information	4	4-4
				Quantity of information	4	4-5
				Visual information	4	4-4
				Credibility of source	4	4-5
Subjective quality	3.14	62.8	0.74	Would recommend the app	4	3-5
				Would use the app in the next 12 months	3	2-3
				Would buy the app	3	1-3
				Overall star rating	4	3-4

^a^IQR: interquartile range.

No significant (*t*_169_=0.67, *P*=.50) between-sex difference emerged in the MARS total scores (mean 4.00, SD 0.40 for men and mean 3.96, SD 0.47 for women). There was a weak positive (*r*=.16, 95% CI .01-.30) correlation between the score and the age of participants. Non-health care professionals scored significantly (*t*_169_=5.59, *P*<.001) higher than health care professionals (4.20, SD 0.32 vs 3.87, SD 0.46), and the effect size was large (*d*=0.92, 95% CI 0.58-1.26). Table 4 reports the final multivariable model. The main effect of respondents’ professions was a significant (*P*<.001) predictor of the MARS total score: on average, health care professionals attributed 0.78 (15.6%) fewer points than those outside the health care sector. Moreover, there was a significant (*P*=.01) interaction between age and profession: with increasing age, health care workers awarded higher scores, while no age-related pattern emerged among non-health care professionals. The model explained 15.5% of variance; its residuals were normally distributed (Shapiro-Wilk test: *P*=.94) but heteroskedastic (Breusch-Pagan test: *P*=.02), justifying the use of robust standard errors. The 5 subscale-specific models (Multimedia Appendix 1) yielded very similar results.

**Figure 5:**
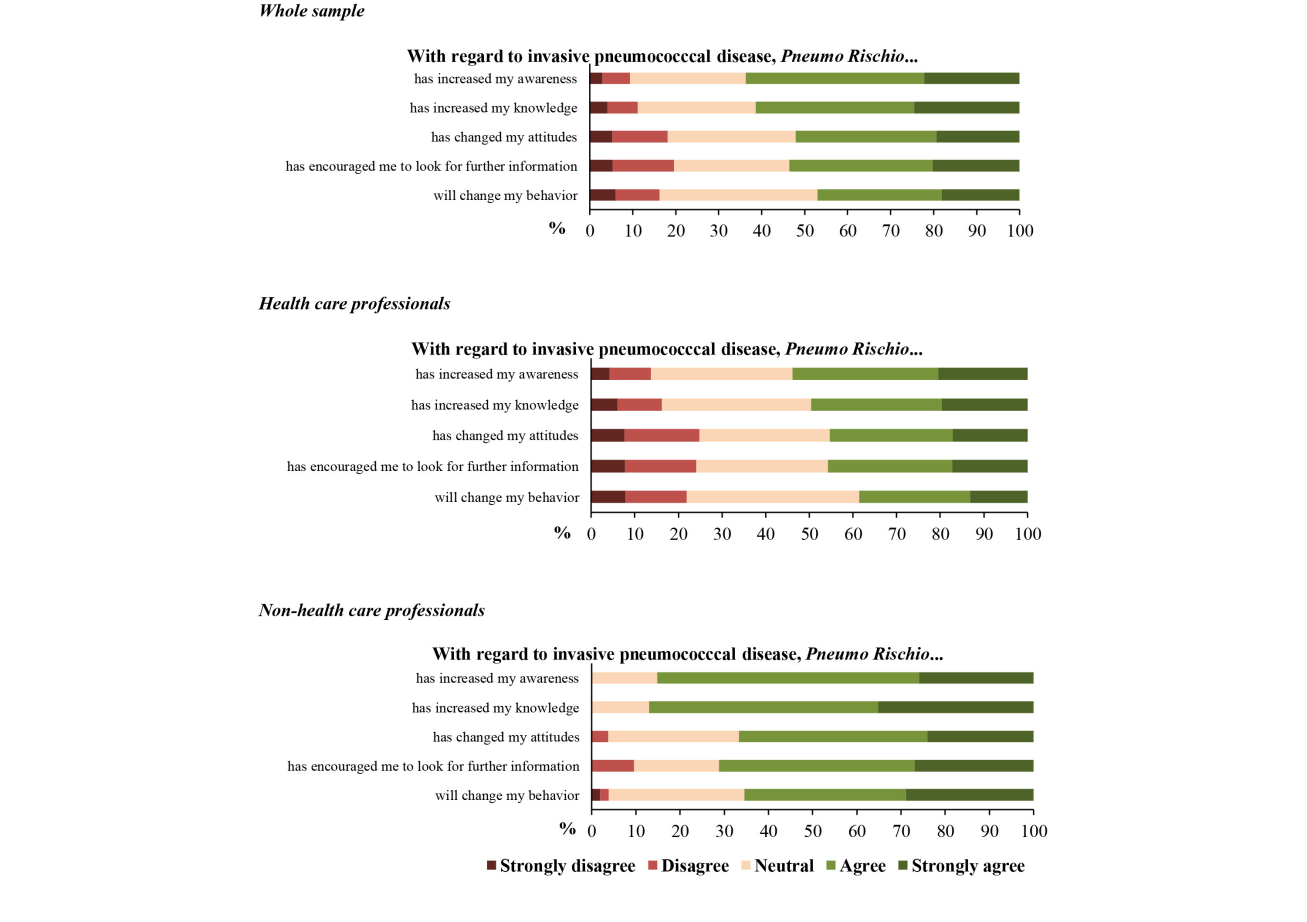
Response patterns on the app-specific user version of the Mobile Application Rating Scale items.

Figure 5 shows response patterns to the app-specific MARS items. More than 60% of respondents attributed a score of at least 4 (ie, “agree” or “strongly agree,” which correspond to the light green and dark green areas of the bars in Figure 5) on items regarding app-induced increase in awareness (109/171, 63.7%) and knowledge (105/171, 61.4%), while these proportions were 52.0% (89/171) for items regarding the app’s ability to modify attitudes, 53.6% (90/168) for items regarding encouragement of a search for further information, and 47.0% (78/166) for items regarding behavior change. However, like the results of the regression analysis, the perceived usefulness of the app differed by professional category. Comparison of the response categories (ie, strongly agree/agree vs neutral/disagree/strongly disagree) revealed that health care professionals assigned markedly lower scores than did people outside the health care sphere on all items.

**Table 4:** Final multivariable linear model to predict the Mobile Application Rating Scale total score.

Predictor	*b* (SE)	| *t*_165_| (*P* value)
Intercept	4.31 (0.13)	32.43 (<.001)
Sex (female vs male)	–0.01 (0.07)	0.21 (.84)
Age	0.23 (0.24)^a^	0.92 (.36)
Profession (health care vs non-health care)	–0.78 (0.20)	3.97 (<.001)
Age × profession	1.36 (0.50)^a^	2.72 (.01)

^a^Estimates are multiplied by 100.

## Discussion

### Principal Findings and Comparison With Previous Work

The main novelty of our study is that it took a multidimensional approach to evaluating an eHealth project. The variety of techniques used gave us an opportunity to analyze *Pneumo Rischio* from the point of view of developers, end users, and public health authorities. However, as we believe that in the eHealth era [39] perspectives of different stakeholders are merging, our discussion will not try to distinguish among single actors.

One of our central findings is that marketing activities and continuous monitoring of usage indicators are fundamental in order to increase population exposure to topic-specific eHealth projects such as *Pneumo Rischio*. According to the developers of the Canadian national immunization app [24], the diffusion of an app is a crucial parameter of its success, but this may be challenging. Although the *Pneumo Rischio* app is cost- and registration-free, contains no in-app advertisements, is available in all main Italian app stores, and was developed with the active involvement of experts in pneumococcal diseases (features potentially associated with greater success [40-42]), daily monitoring of the project usage parameters immediately after its launch prompted us to sketch a marketing mix. Indeed, we judged the preintervention number of downloads (n=340) and website visits (n=3994) [14] to be insufficient to reach the main project goal.

We noted that the 1-year life cycle of *Pneumo Rischio* was clearly divided into two stages, a fact undoubtedly attributable to the promotional campaign. Thus, promotional activities were essential to disseminate information on the project. Indeed, without this intervention, the daily number of *Pneumo Rischio* app users would probably not have changed, or may even have decreased, while implementation of the marketing strategy increased app downloads exponentially. Moreover, the creation of a sister website and social media activity may be valuable ways of increasing population exposure.

Our promotional campaign may be regarded as a natural experiment with an interrupted time series design. This quasi-experimental approach is very robust in quantifying the longitudinal effects of an intervention [33]. The results of the segmented regression analysis will allow us both to forecast the effects of future promotional activities and to compare the effects of chronologically distant interventions. Indeed, segmented regression models allow specifications with more than one change point [33].

We observed that the usage parameters of the app and website differed substantially. Indeed, while the *Pneumo Rischio* website had 15.5 times more visitors than the number of app downloads, an average session was much shorter. This finding supports the results obtained by Hearn et al [43], who suggested that an app and a website should be regarded as complementary resources. These authors concluded that an app may help to engage users, while a website is a useful information source [43]. A higher level of engagement (in that downloading an app requires more effort than visiting a website) may partly explain the longer duration of app sessions.

The Photo type of Facebook posts had a significantly higher number of engaged users. This confirms previous findings that these types of posts enhance user interaction and user engagement, and may be seen as a useful metric for social media marketing [30]. By contrast, we did not confirm our hypothesis that Facebook posts containing personal or possessive pronouns or adjectives (eg, “me,” “my”) engaged more users. Although it has been amply demonstrated that personalized messages are more suitable in terms of people’s engagement [44,45], a purely linguistic approach through the use of personal determiners is probably insufficient. A higher level of customization may therefore be more fruitful.

It should, however, be borne in mind that, although social networking helped to promote the app, it may also engender some risks. For instance, about 2 months after its launch, *Pneumo Rischio* was cited and criticized by an antivaccination Facebook group, “Autism and Vaccines,” which as of September 2016 had more than 8600 fans. Specifically, their post questioned “the eulogized scientific method” and urged people “not to fall into the trap of confusing pneumococcus with meningococcus”—presumably a reference to the causative agent of meningitis. This post is typical of the antivaccination movement; skewing science is a frequent claim of antivaccine activists in the era of Web 2.0 [46]. Moreover, the post used the word trap (*tranello*), which has a clear negative connotation. Although this post had no negative impact on the image of *Pneumo Rischio* or its daily downloads (no negative reviews or decrease in the number of users were registered around that week), this type of risk is difficult to detect and manage.

As expected, in terms of age and sex, *Pneumo Rischio* users were not fully representative of Italian adult Web users [47]. Indeed, most app users and Facebook fans were female, while in the Italian context male Internet users are slightly prevalent. On the other hand, the sex distribution of the website users was very close to that of the reference population. With regard to age, we observed different patterns among app and website users and Facebook fans. More than half of app users were adults aged 25-44 years, while 61% of the website visitors were 18-34 years old. Facebook fans were somewhat older, in that three-quarters were 35- to 64-year-olds. Only the distribution of app users over 35 years of age was close to the reference population of adult Web users.

A noteworthy result regards the distribution of IPD risk categories: about two-thirds of users could be defined as being at risk of IPD. While, to the best of our knowledge, there are no Italian data on IPD risk distribution, a large German study [35] found a significantly lower proportion of people at risk of IPD in the general population. In our opinion, several factors may have contributed to the phenomenon observed. First, individual users might have engaged in multiple sessions. For example, they could have given truthful answers (in order to discover their own risk) when filling in the checker the first time, and could subsequently have answered hypothetically (eg, “What would happen if I had…?”). To address this shortcoming, we considered only single events displayed by Google Analytics. A second explanation may lie in the self-diagnoses made by the app users. We tried to prevent this by wording questions in the third person (“Has your doctor ever told you…?”). However, considering the high specificity of the topic, we believe that the most probable cause of the higher risk in our population is linked to the overrepresentation of users who are really at risk of IPD and were able to locate the website or download the app. Indeed, people with any chronic disorder are more likely than healthy individuals to have a health-related app on their mobile phones [48].

The observed patterns of the distributions of app users in terms of sex, age, and IPD risk categories are consistent with the results from an Italian survey on eHealth use [49]. Indeed, Siliquini et al [49] found a higher probability of using the Internet for health-related purposes among females, younger people, and people with chronic conditions. Interestingly, in that study most male eHealth users were young adults aged 18-29 years, while this proportion was highest among females aged 30-41 years. Although males account for a higher proportion of mobile phone owners [50], females are more likely to install a health-related app [48].

While users of the app, website, and Facebook page were not fully representative of Italian Internet users in terms of age and sex, it is largely unknown who downloads vaccination- or disease-specific apps and surfs the Web for immunization-related purposes. To better understand issues concerning the representativeness of our data, it is worth comparing usage patterns of the *Pneumo Rischio* website with those of *VaccinarSì* [51], which is one of the largest immunization-related Web portals in Italy. The results of a 2-year usage study of the *VaccinarSì* project have recently been published [52]. The *Pneumo Rischio* and *VaccinarSì* websites had a similar share of male and female users, a pattern that reflects the national use of the Internet by both sexes, in which males are slightly prevalent. This phenomenon, which still persists even in developed societies, is known as the digital gender divide [53]. Age-class distributions were very similar among users of both websites, while the Facebook fans of *Pneumo Rischio* were older than those of *VaccinarSì*. Another similarity between the two websites regards the mean duration of sessions and the number of pages viewed [52].

It is encouraging that about 5000 *Pneumo Rischio* users declared their intention to request further information on IPD from their physician, and about 2000 sent an email (probably to their GPs) with their detailed IPD risk profile. In the modern digital age, doctor–patient relationships change continuously and become more participatory when a better-informed patient is more closely involved in the decision-making process [54]. In a large representative sample of US physicians, 85% of interviewees had at least one patient who had brought Web-acquired health-related information to a visit in order to ask the doctor’s opinion on the matter in question [55]. The role of GPs is therefore crucial in enhancing public awareness of IPD and its prevention, especially among so-called vaccine-hesitant people.

To date (as of September 2016), to the best of our knowledge, our investigation is the largest study to have used the MARS in the community setting. Users rated the app as highly functional; that is, highly performing, easy to use and to navigate, and informative (both qualitatively and quantitatively). These 2 MARS subscales exceeded 80%. Indeed, throughout the process of app development [14], the app’s usability properties and its easily comprehensible and evidence-based content were our priority. On the other hand, the subjective quality dimension of the MARS displayed a relatively low mean score, only slightly exceeding 60%. This score was probably lowered by item 19, which concerned the potential purchase of the app; this was the only score with an IQR of 1-3. This may, therefore, suggest that highly targeted and disease-specific apps (especially in the case of diseases with a relatively low incidence) should be free of charge. Indeed, free apps are downloaded much more frequently than paid-for apps, and it has been forecast [56] that, in 2016, 94% of apps will be downloaded free of charge. It is also encouraging that the engagement subscale received a mean score of 75%, since, in addition to the quality of information, interactivity is an important factor that contributes to improving customers’ intention in credence goods or services [57].

Health care professionals attributed lower MARS scores to all subscales and app-specific items. The adjusted regression models to predict both MARS total and subscale scores confirmed this finding. It is, therefore, plausible that health care professionals have higher expectations of a health-related app. These lower scores are not surprising, since the app target was the general adult population [14]. However, research has suggested that only half of physicians actually know the term IPD [11]. *Pneumo Rischio* would therefore be useful to many GPs, too.

### Study Limitations

In interpreting our results we noted three main limitations. First, we were compelled to use proxy measures of project effectiveness, namely intentions to ask GPs for further information on IPD and number of emails with detailed IPD risk profiles sent. In our opinion, these two indicators approximated project effectiveness better than the number of app downloads, website visits, or Facebook fans. An optimal indicator of *Pneumo Rischio* effectiveness would be the proportion of users who really asked their GP for information on IPD and its prevention, or even were vaccinated against pneumococcus after (and because of) using the project. However, obtaining such data is computationally, economically, and ethically challenging. Second, despite the fact that the professional and user versions of MARS are very similar, only the professional Italian version of MARS has been validated. Considering that the two original English versions of MARS differ only slightly (although the user version does not require training), we supposed that the psychometric properties of the professional Italian version of MARS [36] would be similar to those of the user version. Third, as our sample of participants who filled in the MARS was not representative of the population of Italian adult Internet users, our estimates could differ from the average scores of the reference population. Indeed, given that our convenience sample overrepresented health care professionals, who attributed significantly lower MARS scores than the target population of laypeople, the summary MARS scores could be even higher.

### Conclusions

Despite its highly specific topic and somewhat niche nature, *Pneumo Rischio* may be deemed a successful project, as it attracted more than 150,000 users in a 1-year period. Moreover, it was also professionally recognized in an eHealth contest. We therefore hope that our project will contribute to the fight against invasive bacterial diseases.

However, in order to reach their potential end users, such projects should be popularized. Indeed, the process of development of a health-related app should be continuous, and not end with the public release of the app. In our opinion, there is a need to develop a multidimensional framework for assessing health-related apps; this should at least include (1) an evidence base, (2) objective and subjective quality, (3) usefulness, (4) usage indicators, and, if applicable, (5) an outcome assessment.

## Appendix

Multimedia Appendix 1 Final multivariable linear models to predict Mobile Application Rating Scale mean subscale scores.

## Abbreviations

GP: general practitioner
IPD: invasive pneumococcal disease
IQR: interquartile range
MARS: Mobile Application Rating Scale
