# Supplementary material for: An eHealth Project on Invasive Pneumococcal Disease: Comprehensive Evaluation of a Promotional Campaign
Source: J Med Internet Res. 2016 Dec 2;18(12):e316. doi: 10.2196/jmir.6205 (PMC5161781; doi:10.2196/jmir.6205)
Supplement: Multimedia Appendix 1 [file jmir_v18i12e316_app1.pdf]

Multimedia Appendix 1. Final multivariable linear models to predict MARS mean subscale scores.

**Subscale**	**Predictor**	***b* (SE)**	**|*t*| (*P*)**	R^2
Engagement	Intercept	4.17 (0.15)	28.31 (<.001)	.17
	Sex (females vs males)	-0.02 (0.06)	0.27 (.79)	
	Age	-0.43 (0.28)[a]	1.56 (.12)	
	Profession (healthcare vs non-healthcare)	-0.89 (0.20)	4.40 (<.001)	
	Age x profession	1.61 (0.53)[a]	3.06 (.003)	
Functionality	Intercept	4.53 (0.16)	29.17 (<.001)	.06
	Sex (females vs males)	-0.03 (0.09)	0.29 (.77)	
	Age	-0.36 (0.33)[a]	1.10 (.27)	
	Profession (healthcare vs non-healthcare)	-0.68 (0.24)	2.80 (.01)	
	Age x profession	1.36 (0.64)[a]	2.13 (.04)	
Aesthetics	Intercept	4.22 (0.19)	21.71 (<.001)	.11
	Sex (females vs males)	-0.03 (0.09)	0.33 (.74)	
	Age	-0.43 (0.36)[a]	1.20 (.23)	
	Profession (healthcare vs non-healthcare)	-0.85 (0.26)	3.20 (.002)	
	Age x profession	1.48 (0.67)[a]	2.19 (.03)	
Information	Intercept	4.27 (0.14)	29.85 (<.001)	.16
	Sex (females vs males)	-0.01 (0.08)	0.11 (.92)	
	Age	0.31 (0.31)[a]	1.00 (.32)	
	Profession (healthcare vs non-healthcare)	-0.68 (0.23)	3.01 (.003)	
	Age x profession	0.99 (0.60)[a]	1.65 (.10)	
Subjective quality	Intercept	3.74 (0.27)	13.81 (<.001)	.17
	Sex (females vs males)	0.09 (0.11)	0.75 (.45)	
	Age	-0.69 (0.48)[a]	1.44 (.15)	
	Profession (healthcare vs non-healthcare)	-1.46 (0.34)	4.28 (<.001)	
	Age x profession	2.64 (0.85)	3.09 (.002)	

[a]Estimates are multiplied by 100
